# Development, Characterization, and Stability Evaluation of the Anti-Cellulite Emgel Containing Herbal Extracts and Essential Oils

**DOI:** 10.3390/ph14090842

**Published:** 2021-08-25

**Authors:** Ngamrayu Ngamdokmai, Kornkanok Ingkaninan, Nattiya Chaichamnong, Krongkarn Chootip, Nitra Neungchamnong, Neti Waranuch

**Affiliations:** 1 Centre of Excellence in Cannabis Research, Department of Pharmaceutical Chemistry and Pharmacognosy, Faculty of Pharmaceutical Sciences and Center of Excellence for Innovation in Chemistry, Naresuan University, Phitsanulok 65000, Thailand; ngamrayun59@nu.ac.th; 2 Division of Applied Thai Traditional Medicine, Faculty of Public Health, Naresuan University, Phitsanulok 65000, Thailand; nattiyach@nu.ac.th; 3 Department of Physiology, Faculty of Medical Sciences, Naresuan University, Phitsanulok 65000, Thailand; krongkarnc@nu.ac.th; 4 Science Laboratory Centre, Faculty of Science, Naresuan University, Mueang, Phitsanulok 65000, Thailand; nitran@nu.ac.th; 5 Department of Pharmaceutical Technology, Faculty of Pharmaceutical Sciences and Center of Excellence for Innovation in Chemistry, Naresuan University, Phitsanulok 65000, Thailand; 6 Cosmetics and Natural Products Research Center, Faculty of Pharmaceutical Sciences, Naresuan University, Phitsanulok 65000, Thailand

**Keywords:** topical formulation, anti-cellulite, cosmetic, monoterpenoids, accelerated stability

## Abstract

Recently, the herbal compress was successfully developed and applied for cellulite treatment. The aim of this study was to formulate a more convenient dosage form of herbal application from the original formula. In addition, we aimed to characterize and evaluate the stability of the developed dosage form. A gelled emulsion, or an “emgel,” incorporated with 0.1 wt% tea and coffee extracts (1:1 ratio) plus 5 wt% essential oils (mixed oil) was prepared. The caffeine content in the finished product obtained from tea and coffee extracts analyzed by HPLC was 48.1 ± 2.3 µg/g. The bio-active marker monoterpenes of mixed oil characterized by headspace GCMS were camphene 50.8 ± 1.8 µg/mg, camphor 251.0 ± 3.2 µg/mg, 3-carene 46.7 ± 1.8 µg/mg, α-citral 75.0 ± 2.1 µg/mg, β-citral 65.6 ± 1.3 µg/mg, limonene 36.8 ± 6.7 µg/mg, myrcene 53.3 ± 4.5 µg/mg, α-pinene 85.2 ± 0.6 µg/mg, β-pinene 88.4 ± 1.1 µg/mg, and terpinene-4-ol 104.3 ± 2.6 µg/mg. The stability study was carried out over a period of 3 months at 4, 25, and 50 °C. The caffeine content showed no significant changes and passed the acceptance criteria of ≥80% at all tested temperatures. However, monoterpenes showed their stability for only 2 months at 50 °C. Therefore, the shelf-life of the emgel was, consequently, calculated to be 31 months using the Q10 method. Thus, the anti-cellulite emgel was successfully formulated. The characterization methods and stability evaluation for caffeine and monoterpenes in an emgel matrix were also successfully developed and validated.

## 1. Introduction

Cellulite, popularly called orange peel skin, affects mostly women and is found in more than 80% of women of post-pubertal age, which can be treated either medically or cosmetically to enhance the appearance of this issue [1]. Natural ingredients are preferable in cosmetic treatment due to their safety image [2]. Several studies have shown that various natural components are clinically effective [3,4,5,6]. These indicate that plants are a particularly rich source of providing components for cellulite treatment. Previously, anti-cellulite treatment by applying a warmed traditional herbal compress comprising aromatic herbs, i.e., ginger (*Zingiber officinale* Roscoe), black pepper (*Piper nigrum* L.), java long pepper (*Piper retrofractum* Vahl.), plai (*Zingiber montanum* (J. Koenig) Link ex A.Dietr.), turmeric (*Curcuma longa* L.), lemon grass (*Cymbopogon citratus* DC. Stapf.), and kaffir lime (*Citrus hystrix* DC.), and herbs containing xanthine alkaloids, i.e., tea (*Camellia sinensis* (L.) Kuntze) and coffee (*Coffea arabica* L.), on the cellulite area was proven to be effective [7,8]. The anti-cellulite ingredients were considered for their prospective complementarities in combating the cellulite problem. Caffeine [9] (in tea and coffee), limonene [10] (in ginger, black pepper, java long pepper, lemon grass, and kaffir lime), citral [11] (in black pepper, java long pepper, lemon grass, and kaffir lime), and terpinene-4-ol [12] (in plai) among others are cosmetic agents with well-documented anti-adipogenesis action.

The anti-cellulite mechanisms of the essential oils, including monoterpenoids, constituents of aromatic herbs as well as extracts of tea and coffee, have also been elucidated in vitro as a reduction in lipid accumulation and vasorelaxation [13]. Therefore, the active formulation of a smooth-texture gelled emulsion, or an “emgel,” with a pH range of 5.5–7.0 included a combination of essential oil and extract actives, considered for their way of treating all the major mechanisms related to the development of cellulite. Recently, the more convenient dosage form, anti-cellulite emgel, was freshly prepared and clinically tested. Its effectiveness was already proved and reported [14].

The aim of this study was to demonstrate headspace gas chromatography/mass spectrometry (HS-GCMS) and high-performance liquid chromatography (HPLC) methods for monoterpenoid and caffeine determination for the main constituents in the anti-cellulite emgel matrix and evaluate product stability after storage over a period of 12 weeks at 4, 25, and 50 °C.

## 2. Results and Discussion

### 2.1. Anti-Cellulite Emgel Formulation

Many active ingredients from medicinal plants, primarily in the form of standardized botanical extracts, can act synergistically on various biological targets and improve unwanted signs and symptoms [15]. The main ingredients of this formulation were mixed essential oils from well-documented anti-inflammatory and anti-lipogenesis or lipolysis herbs used in our herbal compress, such as ginger [16,17], black pepper [18,19], java long pepper [20,21], and lemon grass [22,23], and water extracts of tea [24,25] and coffee [26,27].

Anti-cellulite emgels were formulated by using a carbomer (Carbopol 940) combined with an emulsion to create a delivery base for hydrophobic substances. The carbomer-to-emulsion ratio was optimized to accommodate the hydrophobic actives. A mixture of seven essential oils of herbal ingredients in the compress (mixed oil) and tea and coffee extracts were chosen as active ingredients of the anti-cellulite formulation. The problem of water insolubility, high volatility, and instability of the short-chain hydrocarbon molecules of the essential oils (e.g., α-pinene, camphene, myrcene, and terpinen-4-ol), being incompatible with the emulsion droplets in the formulation [28], was resolved by introducing a carrier oil with a long lipid tail into the emulsion system as a diluent for the essential oils. The carrier oil also enhanced the viscosity of the oil combination and promoted the development of the emulsion. The carrier oil for this emulsion system was virgin cold-pressed rice bran oil. This mixed oil and two extracts were successfully incorporated into the preliminary developed stable emgel base. The formulation is shown in Table 1.

Texture, color, odor, pH (at 25 °C), and viscosity (spindle no. 5, 20 rpm, 25 °C) of the formulation were observed during the stability tests. Measurements were taken before and after 24 days of storage at 4 °C, followed by six cycles of heating and cooling at 4 and 45 °C for 48 h per cycle. A formulation kept at 25 °C was used as a control.

### 2.2. Chemical Characterization of the Anti-Cellulite Emgel

HS-GCMS was used for qualitative and quantitative analyses of volatile substances in the anti-cellulite emgel. The advantage of headspace analysis over standard GC methods is the ability to determine only volatile analytes, without concern for other matrix components. Headspace sampling is therefore suitable to determine volatile compounds in our emgel or other semisolid cosmetic products. Moreover, the headspace GCMS method provides several advantages, including no use of organic solvents, potential automation, and the ease of sample preparation [29,30]. Twenty-nine constituents of the anti-cellulite emgel were characterized by HS-GCMS (Table 2). The monoterpenes from the essential oil constituents were detected as major constituents. The rank by %peak area was camphor (100.0%), δ-Curcumene (21.6%), limonene (8.8%), β-sesquiphellandrene (8.6%), α-curcumene (6.6%), β-bisabolene (6.4%), sabinene (6.3), tumerone (5.8%), α-citral (5.2%), and β-citral (4.6%).

### 2.3. HS-GCMS Method Validation

#### 2.3.1. Selectivity

**Figure 1:**
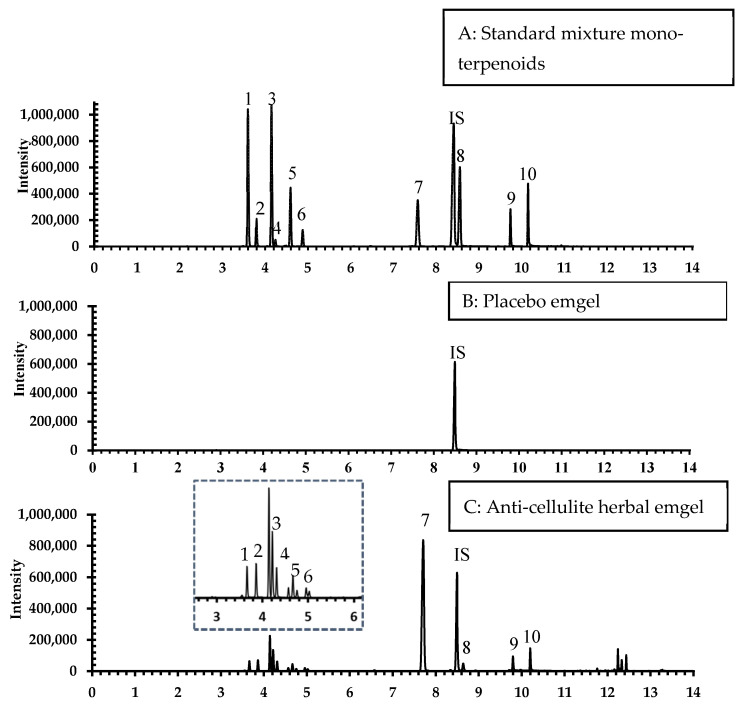
HS-GCMS (SIM) total ion chromatograms of (**A**) standard monoterpenoid mixture (2.5 µg/mL), (**B**) placebo emgel (20 mg), and (**C**) anti-cellulite herbal emgel. The peaks of 10 constituents were identified by comparison with standard references as (1) α-pinene (RT 3.61), (2) camphene (RT 3.81), (3) β-pinene (RT 4.16), (4) myrcene (RT 4.25), (5) 3-carene (RT 4.61), (6) D-limonene (RT 4.89), (7) camphor (RT 7.59), (8) terpinene-4-ol (RT 8.55), (9) β-citral (RT 9.75), and (10) α-citral (RT 10.16).

These values demonstrate that no interfering peaks occurred in the corresponding retention time of each analyte (Figure 1). The selected ion mode (SIM) and retention time (RT) for compounds of interest in HS-GCMS analysis were (1) α-pinene (specific *m/z* ratios: 91, 92, 93), RT 3.611; (2) camphene (specific *m/z* ratios: 79, 93, 121), RT 3.810; (3) β-pinene (specific *m/z* ratios: 69, 91, 93), RT 4.166; (4) myrcene (specific *m/z* ratios: 69, 91, 93), RT 4.257; (5) 3-carene (specific *m/z* ratios: 79, 91, 93), RT 4.605; (6) D-limonene (specific *m/z* ratios: 91, 136), RT 4.895; (7) camphor (specific *m/z* ratios: 69, 81, 95), RT 7.588; (7) menthol (specific *m/z* ratios: 71, 81, 95), RT 8.420; (8) terpinen-4-ol (specific *m/z* ratios: 43, 71, 111), RT 8.560; (9) β-citral (specific *m/z* ratios: 41, 69, 94), RT 9.747; and (10) α-citral (specific *m/z* ratios: 69, 84, 94), RT 10.16.

#### 2.3.2. Linearity

The range of linearity of the 10 constituents with their LOD and LOQ are shown in Table 3. The *r*^2^ for the calibration curves for all compounds was >0.990. For all substances, the signals were linear over concentration ranges, suggesting that the method was appropriate for analyzing these compounds in the same sample.

#### 2.3.3. Precision and Accuracy

Intra-day and inter-day precision and accuracy at three concentrations were studied. Both precision and accuracy were within reasonable limits (%RSD was less than 15%, and percentage accuracy was between 85 and 110%) [31] (Table 4).

The accuracy was determined by spiking the three different concentrations of 10 monoterpenoid standards. Recovery in the range of 94–107 (%RSD ≤ 4.21%) was obtained (Table 4).

### 2.4. HPLC Method Validation

The HPLC method for determination of caffeine was developed and validated according to ICH guidelines.

#### 2.4.1. Selectivity

**Figure 2:**
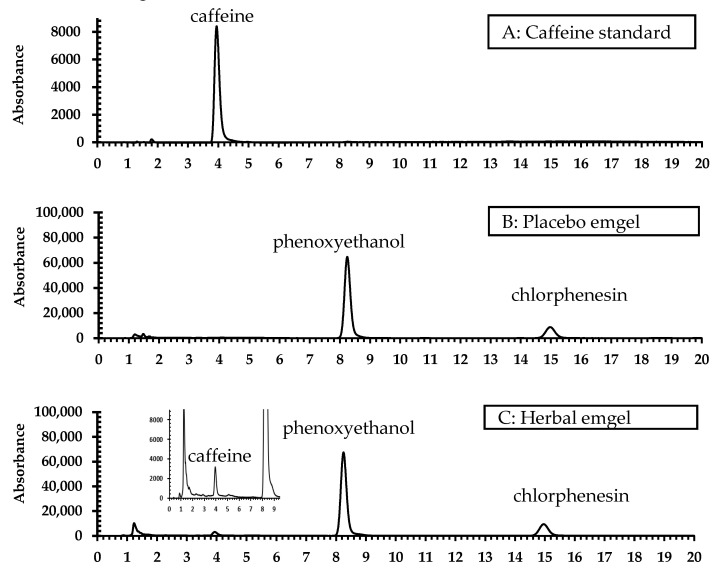
HPLC chromatograms of (**A**) caffeine standard (2.5 µg/mL) (RT 4.015), (**B**) placebo emgel (20 mg/mL), and (**C**) anti-cellulite emgel (20 mg/mL).

Separation was done on a C18 column with isocratic elution of 40% methanol in water. The tailing factor and resolution of the caffeine standard met ICH guidelines. The identification of caffeine in the sample was confirmed with the retention time of the caffeine standard (Figure 2).

#### 2.4.2. Linearity

The calibration equations, linearity, limit of detection (LOD), and limit of quantitation (LOQ) values are presented in Table 5. The HPLC chromatograms of the caffeine standard, placebo, and anti-cellulite emgel are shown in Figure 2A–C.

#### 2.4.3. Precision and Accuracy

Intra-day and inter-day precision and accuracy at three concentrations were determined. Both precision and accuracy were within reasonable limits (% RSD was less than 15%, and percentage accuracy was between 85 and 110%) (AOAC, 2012). (Table 6. The %RSD, which reflected the intra-day and inter-day precision of the caffeine standard, was not more than 2.5%.

The accuracy was determined by spiking the two different concentrations of caffeine standard (1.8 and 7.5 ppm) at 0.1 mg/mL of coffee extract. Recovery in the range of 98–103 (%RSD ≤ 6.32%) was obtained. All data are shown in Table 6.

### 2.5. Quantitation of Constituents of Interest in the Anti-Cellulite Emgel Using HS-GCMS

The 10 constituents of interest were analyzed in the anti-cellulite emgel by HS-GCMS (SIM) (Table 7). The major monoterpene in the formula was camphor (251.0 µg/mg).

### 2.6. Determination of Caffeine Content in Tea, Coffee Material, and the Anti-Cellulite Emgel by HPLC

The caffeine concentrations of coffee, tea, and anti-cellulite emgel were analyzed by HPLC. For the anti-cellulite emgel, a content of 0.05% of each tea and coffee extract in the formulation contained approximately 1% of caffeine (Table 8).

### 2.7. Physical Stability of the Anti-Cellulite Emgel

Physical stability is important to consider while maintaining lipophilic chemicals in emulsion formulae and subsequently in cosmetic products to prevent or mitigate deterioration during storage. The anti-cellulite emgel was evaluated for qualities such as color, odor, pH, viscosity, and phase separation. The anti-cellulite emgel had a homogeneous texture with a buttermilk color and a characteristic odor of herbal essential oils. After storage for 12 weeks (Table 9), the physical properties of the anti-cellulite emgel remained similar to baseline, while after a heating-cooling stability study and after storage at 50 °C for 3 months, there was only a minor change in the appearance of the anti-cellulite emgel.

### 2.8. Chemical Stability Evaluation Using GCMS and HPLC

The active monoterpenoids and caffeine in the anti-cellulite emgel were determined using our validated HS-GCMS and HPLC methods. The anti-cellulite emgel was stored for 12 weeks at 4 °C, room temperature, 50 °C.

**Figure 3:**
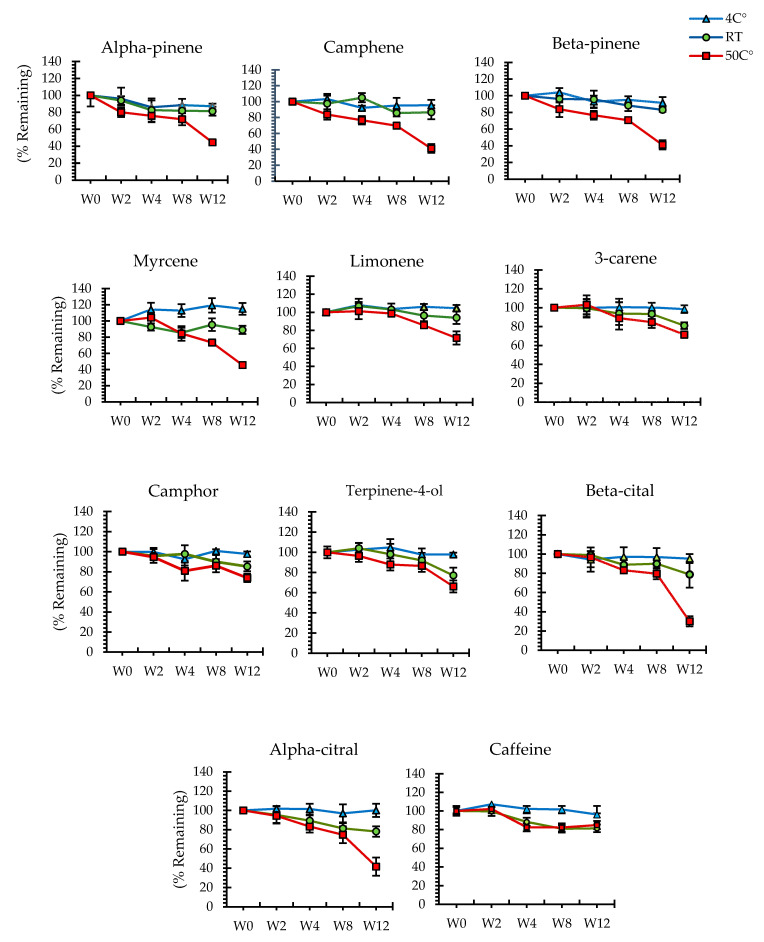
The percentages of the relationship between the time of storage and temperature under room temperature (25 °C), refrigerator (4 °C), and oven (50 °C) conditions. Change in the percentage of constituents of interest, camphor, camphene, citral, 3-carene, limonene, myrcene, α-pinene, β-pinenen, terpinene-4-ol, and caffeine, for 12 weeks.

After 8 weeks storage at 50 °C, the active monoterpenoids in the emgel formulation retained more than 80% of the initiation concentration (Figure 3), whereas caffeine retained more than 80% after 12 weeks (Figure 3).

### 2.9. Microbiological Stability Evaluation

The durability of preservatives is a critical element in ensuring microbial efficiency. To demonstrate microbiological stability, we conducted a preservation challenge test. Our results, shown in Table 10, indicate the acceptance criteria required by the method for acceptability in each time (day 0, day 7, day 14, and day 28). The anti-cellulite emgel was resistant to microbial proliferation, which could pose a risk if used improperly.

### 2.10. Calculation of Monoterpenoids and Caffeine Accelerated Shelf Life by the Q10 Method

The accelerated stability test, which predicts the potential behavior of the anti-cellulite emgel, was quite informative. The shelf life of the anti-cellulite emgel was calculated using the Q10 equation [32,33], which is considered a cornerstone in estimating the storage life of monoterpenoids with heat-sensitive degradation. The storage life of the monoterpenoids in the anti-cellulite emgel was estimated to be 31 months, while that of caffeine was 46 months in the same concentration of the anti-cellulite herbal emgel.

## 3. Materials and Methods

### 3.1. Chemicals and Standards

Methanol and water were of LC-MS grade and purchased from RCI Labscan Ltd., (Bangkok, Thailand). Reference standards, camphor (purity ≥ 95%), camphene (purity ≥ 95%), caffeine anhydrous (purity > 99%), citral (purity ≥ 98%), 3-carene (purity ≥ 90%), limonene (purity ≥ 97%), myrcene (purity ≥ 90%), α-pinene (purity ≥ 98%), β-pinene (purity ≥ 99%), and terpinen-4-ol (purity ≥95%), were products from Sigma-Aldrich (St. Louis, MO, USA). The internal standard, menthol (purity ≥ 98%), was from TCI (Shanghai, China). The standard homologous series of n-alkanes (C8-C40) was obtained from Sigma-Aldrich (St. Louis, MO, USA). Camphor (cosmetic grade) was purchased from Chemipan (Bangkok, Thailand).

### 3.2. Essential Oils and Extracts

Essential oils of ginger, black pepper, java long pepper, plai, turmeric, lemon grass, and kaffir lime were purchased from Thai-China Flavours and Fragrances Industry Co., Ltd. (Ayutthaya, Thailand). They were mixed in a ratio equivalent to the composition of the herbs used in the herbal compress formula [13] and called a “mixed oil.” Tea of the Three Horses brand and roasted coffee of the Arabica 100% Coffman brand were purchased in Phitsanulok, Thailand. Ground tea leaves and coffee beans were separately extracted with boiling water for 20 min. After filtration and 5 min of centrifuging, the aqueous solutions were lyophilized to obtain tea and coffee extracts, which were then stored at −20 °C until use.

### 3.3. Preparation of the Anti-Cellulite Emgel

The anti-cellulite emgel was composed of 5% mixed oil, 5% camphor, 0.05% tea extract, and 0.05% coffee extract. The other ingredients and their functions are listed in Table 11. Camphor was dissolved in the mixed oil, while tea and coffee extracts were added to the carbopol gel and mixed with other ingredients, resulting in the formation of an emgel. The base sample (without the mixed oil) was prepared with similar materials and under identical conditions for formulation.

### 3.4. Headspace Gas Chromatography/Mass Spectrometry (HS-GCMS) and High-Performance Liquid Chromatography (HPLC) Analyses

The HS-GCMS and HPLC methods for determination of chemical ingredients in the anti-cellulite emgel were developed and validated in terms of the limit of detection (LOD), limit of quantitation (LOQ), linearity, accuracy, and precision [31]. A placebo emgel was used as a blank sample.

#### 3.4.1. HS-GCMS Instruments and Chromatographic Conditions

HS analysis was performed using the Agilent G4556-64000 network. The HS auto sampler Agilent PN 7697A was used to directly introduce samples automatically into the Agilent 7890B Gas Chromatography System-5977B MSD model mass spectrometer. After the vials were pressurized with carrier gas, the emgel HS samples were injected into an HP-5 capillary column (5% phenyl methyl silox) (30 m × 250 μm × 0.25 μm; Agilent 19091S-433). The carrier gas was helium (He) with a constant flow rate of 1.3 mL/min. The GC oven temperature was initially set at 70 °C for 3 min, then increased to 100 °C at a rate of 3 °C/min and held for 3 min, and then increased to 250 °C at a rate of 20 °C/min and held for 1 min, with a total run time of 22 min. Mass spectrometry analysis was carried out using an Agilent mass selective detector model 5977B MSD coupled with the gas chromatograph in selected ion mode (SIM) of specific *m/z* ratios for each of the 10 compounds and one internal standard. The mass spectrometer was operated in electron impact ionization mode (70 eV) with a scan range of 50 to 550 amu.

#### 3.4.2. HS-GCMS Method Validation

The method was validated according to ICH guidelines by determining linearity, LOD, LOQ, precision, and accuracy. The calibration curves were obtained with the average of peak area ratios of three replicates. To find the correlation coefficient (*r*^2^) value, six concentration levels of standard solutions were analyzed. Precision was determined using the repeatability between 6 replicate samples of the placebo emgel and spiked with the concentrations of QC1, QC2, and QC3 of the calibration curve and reported as a coefficient of variation (percentage). The accuracy was calculated as the percentage recovery from three replicates of samples spiked with the same concentration that was used for the determination of precision. Intra-day and inter-day precision and accuracy were performed at three concentrations.

#### 3.4.3. Sample Preparation for HS-GCMS

Mixture standard solutions in methanol at concentrations of 500, 250, 125, 62.5, 31.2, and 15.6 µg/mL (camphene, myrcene) and 2500, 1250, 625, 312.5, 156.2, and 78.1 mg/mL (camphor, α-citral, β-citral, 3-carene, limonene, α-pinene, β-pinene, and terpinen-4-ol) were prepared for generating the calibration curve. Menthol in methanol (2 mg/mL) was used as the internal standard.

For preparing the validation samples, 1 mg of a blank matrix (placebo emgel) and a blank sample (blank vial) were spiked with the internal standard and prepared in a headspace vial of 20 mL and then injected into each batch of samples to demonstrate that there was no cross-contamination and interference during the analysis.

#### 3.4.4. Qualitative Analyses of Constituents in the Anti-Cellulite Emgel Using the HS-GCMS Method

Volatile compounds of the anti-cellulite emgel were analyzed by using the HS-GCMS method, stated above, and identified by comparing their spectra and retention times to those of the standard compounds or to those of the NIST MS search 2.2 library, in addition to comparison with their GC Kováts retention indices (RIs) from the literature. The linear RIs for all components were determined by co-injecting the samples with a solution containing the homologous series of n-alkanes (C8-C20). Caffeine was identified by the retention time of the standard compound.

#### 3.4.5. HPLC Instruments and Chromatographic Conditions

Chromatographic analysis was performed using a Shimadzu SCL-10A HPLC system equipped with a Shimadzu SPD-10A UV/Vis detector, an LC-10AT pump, SIL-20AC HT auto-samplers, and a CTO-10ASVP column oven. Chromatographic separation was performed on a Phenomenex Synergi 4u Hydro-RP 80A column (150 × 4.60 mm, 4 µm particle size) connected to a Phenomenex C18 (10 × 4.6 mm, 5 µm) guard column that maintained the temperature at 35 °C. The isocratic mobile phase was methanol and water (40:60 *v/v*) at a flow rate of 1.0 mL/min. The injection volume was 10 µL, and the eluates were monitored at 275 nm. The total run time was 8 min.

#### 3.4.6. HPLC Method Validation

The method was validated according to ICH guidelines by determining linearity, LOD, LOQ, precision, and accuracy. The linearity range of the standards was determined on seven concentration levels that ranged from 0.3125 to 20 µg/mL. Calibration curves were measured on every analysis day, and each sample was determined in triplicate. The standard curves were plotted by areas under the curve of the caffeine standard. The linearity of the calibration curve was assessed by calculating the coefficient of determination (*r*^2^). The LOD and LOQ under the present chromatographic conditions were determined by injecting the standard solutions until the signal-to-noise ratio of each compound was 3 for LOD and 10 for LOQ. The intra-day precision of the method was analyzed from the measurement of two concentration levels of caffeine for six times within 1 day. Precision is represented by the relative standard deviation (%RSD) calculated by standard deviation/mean × 100. Inter-day precision was validated by measuring the %RSD for three consecutive days at two concentration levels, 1.875 and 7.5 μg/mL (*n* = 3, each level). The accuracy and recovery were determined by spiking the known concentration of the standard solution with the coffee extract to obtain two different concentrations (1.875 and 7.5 μg/mL). These experiments were done in triplicate. The accuracy is presented as percentage recovery of the spiked concentration, which was calculated as [(measured standard concentration – standard concentration in the non-spiked sample)/standard concentration spiked] × 100.

#### 3.4.7. Caffeine Standard Solutions for the HPLC Method

The stock solution of the caffeine standard was freshly prepared by dissolving it in methanol to obtain a concentration of 10 mg/mL. This solution was further diluted with water to create standard calibration curves, LOD, and LOQ. The solutions were then filtered through nylon syringe filters with a 0.45 μm pore size.

### 3.5. Determination of Marker Compounds in the Anti-Cellulite Emgel by HS-GCMS and HPLC

Ten monoterpenoids in the anti-cellulite emgel were quantitatively determined by HS-GCMS, and caffeine was analyzed by HPLC analysis.

#### 3.5.1. Sample Preparation for HS-GCMS

A 1 mg sample of the anti-cellulite emgel was weighed in an HS vial of 20 mL and covered with an aluminum crimp cap with a silicon septum. Next, 10 µL of the internal standard was added to each sample vial.

#### 3.5.2. Sample Preparation for HPLC

A 20 mg sample of the anti-cellulite emgel was weighed in a vial of 1.5 mL, dissolved in methanol, and then vortexed for 1 min. The sample solution was filtered through nylon syringe filters with a 0.45 μm pore size.

### 3.6. Accelerated Stability Study of the Anti-Cellulite Emgel

The anti-cellulite emgel was stored at ambient temperature, 4 °C (±2 °C), and 50 °C (±2 °C). The physical properties, including color, odor, pH, viscosity, and phase separation, as well as chemical markers were determined every 2 weeks for 12 weeks. The heating-cooling cycle test was performed by alternating conditions between 4 °C (±2 °C) for 48 h and 45 °C (±2 °C) for 48 h of each cycle for 6 cycles. The studies were conducted in triplicate.

### 3.7. Shelf Life Prediction by the Q10 Method

The Q10 approach is a tool for forecasting the product shelf life. It is assumed that the ratio of times to equal harm at two temperatures, which are usually 10 °C apart, is constant [32,33,34]. The shelf life of the anti-cellulite emgel at 25 °C was calculated using the following equation:

$$t_{80}(T_{2})=\frac{t_{80}(T_{1})}{Q^{(\Delta T/10}}$$

where *t*_80_ (T2) denotes the shelf life at 25 °C and *t*_80_ (T1) denotes the shelf life at 50 °C.

### 3.8. Microbial Stability Studies

Microbial testing of the anti-cellulite emgel was carried out using a preservation test with the plate method. Five types of microorganisms, i.e., *Staphylococcus aureus* TISTR 1466, *Pseudomonas aeruginosa* ATCC 25783, *Escherichia coli* ATCC 25922, *Candida albicans* ATCC 10231, and *Aspergillus niger*, were added to the anti-cellulite emgel formulation. The criteria of acceptance and the consideration of preservative stability were measured according to USP 29 Chapter 51 Antimicrobial Effectiveness [35].

### 3.9. Statistical Analysis for Quality Control Studies

Data were expressed as the average ± standard deviation (SD). Statistical analysis was conducted using analysis of variance (ANOVA) and Student’s *t*-test using GraphPad.

## 4. Conclusions

An anti-cellulite herbal emgel of smooth texture with pH 6.89 ± 0.02 was successfully formulated. HS-GCMS and HPLC methods were developed and validated to quantitatively determine the monoterpenoid and caffeine constituents in the formulation, respectively. The emgel was physically stable up to 3-month storage at 4 °C, room temperature, and 50 °C. The caffeine content showed no significant changes and passed the acceptance criteria of ≥80% at all temperature tests, while monoterpenes showed some degree of degradation at 50 °C after 2 months. The shelf life of the emgel was, consequently, calculated as 31 months by using the Q10 method.

## 5. Patents

The petit patent for the anti-cellulite herbal emgel product was obtained from the Department of Intellectual Property (DIP), Thailand (no. 17425, date 11 March 2021).

## Figures and Tables

**Table 1:** Anti-cellulite emgel formula.

Ingredient	Formula (wt%)
Deionized water	66.00
Carbopol 940	0.80
Disodium EDTA	0.10
Propylene glycol	2.00
Glycerin	6.00
Phenoxyethanol and chlorphenesin	1.00
Triethanolamine (TEA)	1.00
PEG-40 hydrogenated castor oil	5.00
Rice bran oil	8.00
Tea extract	0.05
Coffee extract	0.05
Mixed oil	5.00
Camphor	5.00

**Table 2:** The volatile composition of the anti-cellulite emgel analyzed by using HS-GCMS.

Peak No.	RT (min)	RI ^1^	Identified Compounds	Relative Area (%)
1	1.66	562	Trimethoxyborane	2.57
2	3.67	939	α-Pinene	2.06
3	3.86	955	Camphene	2.81
4	4.14	978	Sabinene	6.31
5	4.21	984	β-Pinene	4.38
6	4.31	992	Myrcene	2.03
7	4.57	1009	α-Phellandrene	1.14
8	4.67	1014	3-Carene	3.93
9	4.75	1019	α-Terpinene	1.22
10	4.95	1029	Limonene	8.84
11	5.03	1033	Eucalyptol	1.39
12	5.49	1058	γ-Terpinene	1.64
13	6.13	1092	α-Terpinolene	1.55
14	7.71	1152	Camphor	100.00
15	8.49	1180	(Internal standard) menthol	31.01
16	8.63	1185	γ-Terpinene	4.29
17	8.92	1195	α-Terpineol	1.83
18	9.45	1227	(Preservative) phenoxyethanol	7.95
19	9.73	1250	β-Citral	4.60
20	10.19	1277	α-Citral	5.22
21	11.75	1373	Caryophyllene	2.26
22	12.15	1395	α-Curcumene	6.66
23	12.20	1400	Germacrene D	1.87
24	12.23	1404	delta-Curcumene	20.43
25	12.28	1409	β-Selinene	3.15
26	12.32	1413	β-Bisabolene	6.41
27	12.43	1424	β-Sesquiphellandrene	8.46
28	13.25	1511	Tumerone	5.81
29	13.42	1530	Curlone	1.26

^1^ Comparison with Kováts retention index (RI), the NIST library (version 2.2) comparison using GCMS (SCAN) analysis.

**Table 3:** Correlation coefficient (*r*^2^), linear range, LOD, and LOQ of 10 monoterpenoids in the anti-cellulite emgel analyzed by HS-GCMS.

Analytes	RT	(*r*^2^)	Linear Range (µg/mL)	LOD (µg/mL)	LOQ (µg/mL)
α-Pinene	3.611	0.9997	39.1–1250	13.0	39.1
Camphene	3.810	0.9989	62.5–2000	20.8	62.5
β-Pinene	4.166	0.9982	39.1–1250	13.0	39.1
Myrcene	4.257	0.9978	62.5–2000	20.8	62.5
3-Carene	4.605	0.9987	39.1–1250	13.0	39.1
Limonene	4.895	0.9974	39.1–1250	13.0	39.1
Camphor	7.588	0.9989	62.5–2000	20.8	62.5
Terpinene	8.420	0.9964	39.1–1250	13.0	39.1
β-Citral	8.560	0.9988	62.5–2000	20.8	62.5
α-Citral	9.747	0.9976	62.5–2000	20.8	62.5

**Table 4:** Intra-day and inter-day precision and accuracy of the HS-GCMS method for determination of 10 monoterpenoid standards assessed at three different concentration levels (*n* = 3) on three consecutive days. Accuracy was expressed as the percentage recovery of 10 monoterpenoid standards in three different concentrations in the placebo emgel.

Analytes	Concentration Levels/Spiked Mount (µg/mL)	Intra-Day (*n* = 3)		Inter-Day (*n* = 9)		Accuracy (*n* = 9)
		Measured Concentration (µg/mL) ± SD	Precision (%RSD)	Measured Concentration (µg/mL)	Precision (%RSD)	Recovery (%)
α-Pinene	120	126.62 ± 4.07	3.21	124.45 ± 3.54	2.85	105.5
	190	190.75 ± 1.38	0.73	190.90 ± 1.03	0.54	98.6
	1000	1013.42 ± 5.56	0.54	1004.97 ± 10.63	1.06	98.8
Camphene	125	131.09 ± 2.41	1.84	128.40 ± 3.88	3.02	97.4
	300	284.46 ± 6.33	2.23	277.07 ± 11.66	4.21	101.8
	1600	1561.43 ± 7.74	0.49	1568.28 ± 9.42	0.60	99.4
β-Pinene	120	109.23 ± 3.17	2.91	107.70 ± 2.83	2.63	100.5
	190	192.64 ± 4.88	2.53	193.65 ± 4.11	2.12	100.4
	1000	1013.34 ± 9.99	0.98	1012.33 ± 9.53	0.94	100.0
Myrcene	125	112.72 ± 4.11	3.64	113.48 ± 2.846	2.50	100.6
	300	273.69 ± 3.84	1.40	272.10 ± 8.16	2.99	101.1
	1600	1436.74 ± 4.31	0.30	1442.68 ± 11.36	0.78	106.7
3-Carene	120	124.26 ± 4.42	3.56	122.61 ± 4.22	3.44	94.4
	190	189.28 ± 3.96	2.09	186.85 ± 3.96	2.45	102.0
	1000	924.90 ± 6.15	0.66	927.38 ± 5.33	0.57	102.6
Limonene	120	130.95 ± 3.20	2.44	129.40 ± 2.91	2.91	105.5
	190	194.62 ± 5.02	5.02	191.83 ± 5.34	2.78	102.1
	1000	1025.84 ± 9.62	0.94	1025.34 ± 13.53	1.32	98.3
Camphor	125	134.15 ± 5.09	3.79	134.26 ± 5.09	3.79	98.2
	300	286.33 ± 8.94	3.12	291.26 ±9.32	3.20	96.7
	1600	1718.85 ± 2.75	0.16	1718.85 ± 11.69	0.68	97.8
Terpinene-4-ol	120	114.58 ± 2.17	1.89	112.78 ± 4.19	3.71	100.4
	190	177.21 ± 4.89	2.76	176.40 ± 3.47	1.97	107.5
	1000	942.44 ± 7.19	0.76	934.46 ± 10.47	1.12	101.6
β-Citral	125	107.75 ± 0.95	0.89	108.02 ± 2.31	2.14	98.3
	300	314.81 ± 9.63	3.06	311.76 ± 8.18	2.62	96.2
	1600	1649.47 ± 13.03	0.79	1663.07 ± 14.08	0.85	99.0
α-Citral	125	128.02 ± 4.96	3.88	127.39 ± 4.07	3.19	99.5
	300	329.87 ± 7.54	2.28	329.24 ± 5.74	1.74	99.0
	1600	1642.30 ± 12.91	0.79	1645.17 ± 11.29	0.69	99.0

**Table 5:** Correlation coefficient (*r*^2^), linear range, LOD, and LOQ of the caffeine standard in the anti-cellulite emgel analyzed by HPLC.

Analyte	RT (min)	(*r*^2^)	Linear Range (µg/mL)	LOD (ng/mL)	LOQ (ng/mL)
Caffeine	4.015	1.00	0.3125–20	156.250	15.625

**Table 6:** Intra-day and inter-day precision and accuracy of the HPLC method for determination of the caffeine standard assessed at two different concentration levels (*n* = 3) on three consecutive days. Accuracy was expressed as the percentage recovery of the caffeine standard at two different concentrations in the coffee extract.

Standard	Concentration Levels/Spiked Mount (µg/mL)	Intra-Day Precision (*n* = 6)		Inter-Day Precision (*n* = 9)		Accuracy (*n* = 9)
		Measured Concentration (µg/mL) ± SD	Precision (%RSD)	Measured Concentration (µg/mL) ± SD	Precision (%RSD)	Recovery (%)
Caffeine	1.88	1.86 ± 0.03	1.70	1.81 ± 0.05	2.51	101.0
	7.50	7.49 ± 0.19	2.52	7.32 ± 0.18	2.51	100.6

**Table 7:** The contents of 10 monoterpenoid constituents in the anti-cellulite emgel analyzed by HS- GCMS (*n* = 3).

Anti-Cellulite Emgel	Monoterpenoids Presented in the Formulation (µg/mg) Average ± S.D.
α-Pinene	85.2 ± 0.6
Camphene	50.8 ± 1.8
β-Pinene	88.4 ± 1.1
Myrcene	53.3 ± 4.5
3-Carene	46.7 ± 1.8
Limonene	36.8 ± 6.7
Camphor	251.0 ± 3.2
Terpinene-4-ol	104.3 ± 2.6
β-Citral	65.6 ± 1.3
α-Citral	75.0 ± 2.1

**Table 8:** The content of caffeine in the anti-cellulite emgel was analyzed by HPLC (*n* = 3).

Sample	Caffeine Content Ave ± S.D. (µg/g)
Coffee extract (freeze-dried)	45.0 ± 0.4
Tea extract (freeze-dried)	64.2 ± 1.1
Anti-cellulite emgel with tea and coffee extracts in this formulation	48.1 ± 2.3

**Table 9:** The physical stability of the anti-cellulite emgel at initiation day and after being stored at 4 °C, 25 °C, and 50 °C for 3 months and after 6 heating-cooling cycles.

Conditions	Physical Examination	pH	Viscosity (Cp)	Separation
Initiation	Smooth texture, pale brown	6.89 ± 0.02	1715 ± 5.29	No phase separation
4 °C	Smooth texture, pale brown	6.61 ± 0.04	1681 ± 6.81	No phase separation
25 °C	Smooth texture, pale brown	6.68 ± 0.05	1632 ± 5.50	No phase separation
50 °C	Smooth texture, pale brown (darker, slightly stronger smell)	6.63 ± 0.02	1616 ± 5.03	No phase separation
Heating-cooling 6 cycles 45 °C/4 °C	Smooth texture, pale brown (slightly darker, slightly stronger smell)	6.65 ± 0.03	1654 ± 3.21	No phase separation

**Table 10:** Preservation efficacy of the anti-cellulite emgel.

Microbes	Log_10_ CFU/g			
	Day 0	Day 7	Day 14	Day 28
*Staphylococcus aureus*	6.0	3.3	<2.0	<2.0
*Pseudomonas aeruginosa*	5.9	3.0	<2.0	<2.0
*Escherichia coli*	6.0	<2.0	<2.0	<2.0
*Candida albicans*	4.4	<2.0	<2.0	<2.0
*Aspergillus niger*	4.6	<2.0	<2.0	<2.0

**Table 11:** Anti-cellulite emgel ingredients.

Anti-Cellulite Emgel	Function
Deionized water	Diluent
Carbopol 940	Gelling agent
Disodium EDTA	Chelating agent
Propylene glycol	Moisturizing agent
Glycerin	Moisturizing agent
Phenoxyethanol and chlorphenesin	Preservatives
Triethanolamine (TEA)	pH adjuster
PEG-40 hydrogenated castor oil	Solubilizer
Rice bran oil	Emollient
Tea extract	Active ingredient
Coffee extract	Active ingredient
Mixed oil	Active ingredient
Camphor	Active ingredient

## Data Availability

Data are contained within the article.
